# Complete Genome Sequence of the Hyperthermophilic and Acidophilic Archaeon Saccharolobus caldissimus Strain HS-3^T^

**DOI:** 10.1128/mra.01078-21

**Published:** 2022-03-09

**Authors:** Hiroyuki D. Sakai, Norio Kurosawa

**Affiliations:** a Department of Science and Engineering for Sustainable Innovation, Faculty of Science and Engineering, Soka University, Hachioji, Tokyo, Japan; Indiana University, Bloomington

## Abstract

The complete genome sequence of the hyperthermophilic archaeon Saccharolobus caldissimus strain HS-3^T^ was determined. The genome is 3,075,795 bp with a GC content of 32.9%. Genes for the complete semiphosphorylative Entner-Doudoroff pathway, gluconeogenesis, tricarboxylic acid cycle, pentose phosphate pathway, and 3-hydroxypropionate 4-hydroxybutylate cycle were present in the genome.

## ANNOUNCEMENT

Saccharolobus caldissimus strain HS-3^T^ is a facultatively anaerobic, hyperthermophilic, and acidophilic archaeon belonging to the order *Sulfolobales* (1). It grows at 65 to 93°C (optimal, 85°C) and pH 1.5 to 6.0 (optimal, 3.0) and is capable of utilizing a variety of organic substrates as a carbon source. Although physiological properties of *S*. *caldissimus* were examined in detail (1), the genome sequence of this species has not been determined. Here, we report the complete genome sequence of *S*. *caldissimus* strain HS-3^T^.

Strain HS-3^T^ was isolated as described previously (1). The strain was cultivated in modified Brock’s basal salt medium (2) supplemented with 1 g/L yeast extract (pH 3.0; 80°C). Microbial cells were collected as a pellet by centrifugation (12,000 × *g*, 4°C, and 15 min) from 200 mL of the culture. Genomic DNA was extracted from the cell pellet using Genomic-tips 100/G (Qiagen). Genome sequencing was performed using the same protocol as described previously (3). For short-read sequencing, a DNA library was prepared with the NEBNext Ultra II FS DNA library preparation kit for Illumina (New England BioLabs) and then subjected to an Illumina NovaSeq 6000 sequencer (2 × 150 bp). For long-read sequencing, a DNA library was prepared with kits SQK-LSK109 and EXP-NBD104 (Oxford Nanopore Technologies [ONT]), following the protocol described by ONT (NBE_9065_v109_revZ_14Aug2019). DNA fragments of 3 kb or longer were enriched by the protocol. Long-read sequencing was conducted on a MinION sequencer with an R9 flow cell. Base calling was carried out by MinKNOW v.4.2.8 software. As a result, a total of 8,375,640 short reads (1,256,346,000 bp) and 62,599 long reads (336,859,512 bp [*N*_50_, 15,506 bp]) were obtained. The short reads and long reads were quality filtered using fastp v.0.20.1 (4) and Filtlong v.0.2.0. (https://github.com/rrwick/Filtlong) (Table 1). After the quality-filtering step, a total of 8,322,466 short reads (1,211,007,369 bp) and 56,658 long reads (294,131,198 bp [*N*_50_, 8,652 bp]) were obtained. Using all the quality-filtered reads as the input, genome assembly was carried out with Unicycler v.0.4.8 (5) (Table 1). Genome annotation was conducted by DFAST v.1.4.0 (6). Default parameters were used for all software unless otherwise specified.

**TABLE 1:** Commands used in bioinformatics analyses

Method	Commands
Quality filtering of raw short reads (fastp v.0.20.1)	fastp -i raw-short-read1.fastq -I raw-short-read2.fastq -o quality-filtered-short-read1.fastq -O quality-filtered-short-read2.fastq
Quality filtering of raw long reads (Filtlong v.0.2.0)	filtlong -1 quality-filtered-short-read1.fastq -2 quality-filtered-shortread1.fastq --min_length 1000 --trim --split 100 --mean_q_weight 10 long-read.fastq > quality-filtered-long-read.fastq
Hybrid assembly (Unicycler v.0.4.8)	unicycler -1 quality-filtered-short-read1.fastq -2 quality-filtered-short-read2.fastq -l quality-filtered-long-read.fastq -o assembly-files

As a result of assembly, the complete genome sequence of strain HS-3^T^ was obtained as a circular contig composed of 3,075,795 bp with a GC content of 32.9%. The genome encoded 3,307 coding DNA sequences (CDSs), a single copy of the rRNA operon (16S-23S), 47 tRNAs, and 7 CRISPR repeats. The genome contains genes for complete glycolysis (semiphosphorylative Entner-Doudoroff pathway), gluconeogenesis, tricarboxylic acid cycle, and pentose phosphate pathway. The archaeal carbon fixation pathway 3-hydroxypropionate 4-hydroxybutylate cycle (7) was also found in the genome. Genes related to oligosaccharide transporters (e.g., *malK*, *msmXK*, *smoK*, *msiK*, and *msmX*) and monosaccharide transporters (e.g., *glcSUTV* and *malK*), were present, which was in good agreement with physiological characteristics of HS-3^T^ which utilizes a variety of sugars as a carbon source (1). Based on the full-length 16S rRNA gene, the closest species of HS-3^T^ is Saccharolobus shibatae with sequence similarity of 96.4%, which was calculated using the EZBioCloud 16S-based identifier (ID) (8). Average nucleotide identity (ANI) between HS-3^T^ and *Saccharolobus shibatae* DSM5389^T^ was 74.7%, which was calculated using the EzBioCloud ANI calculator (9).

In this report, we determined the complete genome sequence of the strain HS-3^T^ of *Saccharolobus caldissimus*. The data deposited in the DDBJ/ENA/GenBank database contribute to our understanding of the genomic diversity and evolution of members of the order *Sulfolobales*.

### Data availability.

The genome sequence of strain HS-3^T^ and raw reads have been deposited in DDBJ/ENA/GenBank under the accession numbers AP025226, DRR326939, and DRR326940.
